# Hypromellose-, Gelatin- and Gellan Gum-Based Gel Films with Chlorhexidine for Potential Application in Oral Inflammatory Diseases

**DOI:** 10.3390/gels10040265

**Published:** 2024-04-15

**Authors:** Monika Wojtyłko, Anna Froelich, Barbara Jadach

**Affiliations:** 1 3D Printing Division, Chair and Department of Pharmaceutical Technology, Poznan University of Medical Sciences, 3 Rokietnicka Street, 60-806 Poznań, Poland; 2 Doctoral School, Poznan University of Medical Sciences, 70 Bukowska Street, 60-812 Poznań, Poland; 3 Division of Industrial Pharmacy, Chair and Department of Pharmaceutical Technology, Poznan University of Medical Sciences, 3 Rokietnicka Street, 60-806 Poznań, Poland

**Keywords:** chlorhexidine, hypromellose, gellan gum, gelatin, polymer film

## Abstract

The oral cavity is constantly exposed to contact with an external environment. Pathogens can easily access and colonize it, causing a number of medical conditions that are usually accompanied by inflammation, which in turn require medical intervention and cause the deterioration of wellbeing. The aim of this study was to obtain polymer films that could be a carrier for chlorhexidine, an active substance used in the treatment of inflammation in the oral cavity, and at the same time act as a dressing for the application on the mucous membrane. Combinations of three biocompatible and biodegradable polymers were used to prepare the films. The obtained samples were characterized by assessing their water loss after drying, swelling ability, hygroscopicity and tensile strength. It was shown that the mixture of HPMC and gellan gum or gelatin could be used to prepare transparent, flexible polymer films with chlorhexidine. All tested films showed high hygroscopicity and swelling ability. However, it was observed that the composition containing gellan gum was more suitable for obtaining films with prolonged stay at the site of administration, which predisposes it to the role of a local dressing.

## 1. Introduction

The oral cavity is the first and the most external section of the gastrointestinal tract, and it also communicates with the upper respiratory airways through the pharynx. Except from the obvious barrier function, protecting the distal parts of the gastrointestinal tract from pathogens, the oral cavity plays other important roles, including sensory functions, the secretion of digestive enzymes with saliva, mechanical food processing and verbal communication. The internal surface of the oral cavity is lined with different types of mucous membranes, depending on the region of occurrence. The renewal cycle for oral mucosa cells is estimated at 7 to 14 days [1]. An important part of mouth physiology is its microbiome, which forms a biofilm on dental surfaces. The composition of the oral microbiome is highly complex and may change throughout life, depending on diet, genetic predispositions, medical conditions, applied therapies, hygienic habits and other factors [2]. It is important to notice that the oral cavity is a specific region subjected to a number of changing parameters, like different pathogens, masticatory forces, salivary flow and different food components. As the oral cavity is also exposed to contact with the external environment, pathogens can easily access and colonize it, causing a number of medical conditions usually accompanied by an underlying inflammatory state. The disturbance of microbiological balance in the oral cavity combined with other factors, including poor oral hygiene, impaired immunological responses, smoking or applied chemo- or radiotherapy, may lead to the development of oral mucositis, periodontitis or other conditions that are associated with pain and that negatively affect the general quality of a patient’s life. It must be also emphasized that oral health can be correlated with other aspects of general wellbeing [3]. The bacterial species present in the biofilm formed in the oral cavity can be spread via the bloodstream through the whole body and exert negative effects on different organs. For example, the potential risk of cardiovascular complications associated with periodontal disease was described in the scientific literature [4,5]. As one of the possible explanations, the fact that *Streptococcus mutans* and *Porphyromonas gingivalis*, bacterial strains responsible for the development of caries and periodontitis, induce platelet ag-gregation was mentioned in the literature [6]. Moreover, it was found that hemagglutinins expressed by *P. gingivalis* might contribute to the increased ability to adhere and invade human coronary endothelium cells [7,8]. However, even though the available studies and metaanalyses indicate the correlation between the occurrence of periodontal disease and cardiovascular complications, the question regarding the causative relation between these two remains unanswered [9]. Similar investigations are conducted for the relations between oral inflammation and other conditions, including rheumatoid arthritis, type 2 diabetes, gastrointestinal and colorectal cancer and others [10]. It is noteworthy that periodontitis, as chronic inflammatory condition, reveals some similar features in terms of molecular and cellular mechanisms when compared to autoimmune diseases. Intraoral bacteria can also be responsible for the development of pneumonia upon the saliva aspiration, which is particularly important in the case of elderly patients [11,12]. Apart from microbiome-related imbalance, there are other factors contributing to the development to oral inflammatory diseases. Oral mucositis can occur as a side effect of chemo- and radiotherapy applied in cancer treatment. It is estimated that about 40% of patients receiving chemotherapy in general develop oral mucositis and the risk is about 90% in the case of patients suffering from head and neck cancers treated with chemo- and radiotherapy [13]. It is noteworthy that painful lesions occurring in oral cavity can significantly decrease the quality of patient’s life [14], causing also chewing and swallowing problems that may contribute to general cancer-related malnourishment. In severe cases, hospitalization, tube feeding, chemotherapeutic doses reduction or radiotherapy discontinuation are necessary, which obviously contribute to the diminished efficacy of anticancer treatment [15]. Multinational Association of Supportive Care in Cancer and International Society of Oral Oncology (MASCC/ISOO) published detailed clinical guidelines regarding preventive and therapeutic approaches in cancer-affected patients being at risk of oral mucositis development [16]. On the other hand, oral inflammation is a broad term and comprise also autoimmune diseases, like lichen planus [17], and conditions of unclear etiology, like recurrent aphthous stomatitis [18]. Another manifestation of the inflammatory conditions in the mouth, usually also associated with other underlying conditions including trauma, infections, autoimmune diseases or malignancies, are ulcerative lesions [19]. According to the report recently published by the World Health Organization (WHO) [20], about a half of the global population suffer from different kinds of oral diseases which affect also general health. The most important conclusion of the report is related to the need for preventive actions; however, in the case of already-existing conditions, different therapeutic options are also needed.

Chlorhexidine gluconate (CHX) is a commonly known antimicrobial agent developed first in 1940s and since then widely applied as skin antiseptic. Since 1970s it has been used in oral care to prevent plaque development and also in managing periodontitis syndromes, like bleeding and inflammation [21,22]. It can also be used in medical equipment, like catheters and wound dressings, to prevent catheter-related blood-stream infections [23,24]. It is regarded as a generally well tolerated wide-spectrum antimicrobial agent and, therefore, it is available in a number of marketed formulations applied in oral infections, including mouth rinses and oral gels. CHX can be used both in management and prevention of oral infections, especially in patients unable to maintain proper oral hygiene. In the case of periodontitis patients subjected to non-surgical treatment, sustained release CHX-loaded chips inserted in periodontal pockets (PerioChip®, Dexcel-Pharma, Daventry, UK) are applied [21]. Despite the re-cent concerns related to the potential CHX cytotoxicity [25] and the development of bacterial strains revealing reduced susceptibility to CHX [26], the drug is still very popular and widely applied in dentistry. It must be emphasized that most of the currently marketed formulations reveal instant drug release, while the prolonged release products are limited to intrapocket drug delivery systems, including the mentioned polymer films with CHX, as well as fibers, microspheres and biodegradable gel with other active ingredients [27]. To the best of our knowledge, no sustained release for-mulations intended for the administration to the surface of oral mucosa have been marketed so far. 

As oral inflammation significantly diminishes the quality of life, particularly in patients subjected to different forms of anticancer treatment, a proper therapeutic approach is necessary to prevent further consequences of the disease development. One of novel concepts that can be useful in the management of inflammatory symptoms, especially ulcerative lesions, are hybrid wound dressings composed of materials adhering to oral mucosa and swelling upon the contact with saliva, with incorporated active ingredients released in a controlled manner [28]. The dressings may take the form of tablets or films and must contain mucoadhesive polymers with high ability to swell in an aqueous environment. A swollen polymer matrix occurring as a result of the interaction with saliva can act as a barrier, protecting the affected site from mechanical injuries and irritation related to the food processed in the mouth. On the other hand, the matrix is responsible for slow diffusion of the drug to the lesion and, depending on the system construction, also to the saliva. In this way, the drug concentration can also be delivered in a prolonged way to other areas affected with inflammation.

In this study, novel polymer films composed of hypromellose (HPMC) and gelatin or gellan gum (GG) with CHX as a model antiseptic drug were widely employed in oral formulations were formulated and investigated. HPMC is well-known hydrogel matrix-forming agent frequently incorporated in prolonged release products because of its excellent gelling properties. However, its mucoadhesive properties are considered as moderate [29]. Therefore, additional biopolymers were applied in order to modify the character-istics of HPMC-based matrix. According to the available literature reports, gelatin used as polymer blend component can improve the bioadhesive properties of the obtained system [30,31,32]. On the other hand, gellan gum apart from mucoadhesive characteristics [33,34] can also interact with cations present in the saliva and, therefore, improve the mechanical durability of the films, via the process of ionotropic gelation [35]. The aim of this study was to develop novel CHX-loaded films and analyze their physicochemical properties, including tensile strength, disintegration time, and swelling and moisture absorption ability. The most important objective of the project was to evaluate the impact of the applied film composition and the drug presence on the properties of the final product. 

## 2. Results and Discussion

### 2.1. Formulation Optimization

The aim of this study was to obtain polymer films that could be a carrier for chlorhexidine (CHX), an active substance used in the treatment of inflammation in the oral cavity, and at the same time act as a dressing for application on the mucous membrane. The obtained films should have the ability to adhere to the mucous membrane and remain in contact with it for some time, creating a mechanical protective barrier at the site of inflammation, and then dissolve. Moreover, the matrices should have the ability to swell, allowing the slow release of the active ingredient. Hypromellose 90SH-4000 mPa·s combined with food gelatin or gellan gum with a high degree of acylation was used to obtain a polymer matrix, while deionized water was used as a solvent, and glycerol was employed as a plasticizer. At the preliminary stage, eight combinations of the mentioned components were tested (Table 1). All of the employed polymers have gel-forming ability. Hypromellose was selected due to its bioadhesive properties and good film-forming abilities [36,37,38,39]. The type applied in this study, 90SH, with an average viscosity of 4000 mPa·s, allows for the preparation of swelling polymer matrices used as carriers for prolonged-release drugs [36]. The choice of a polymer with a relatively high molecular weight was dictated by the need to extend the disintegration/erosion time of the matrix, which would also contribute to the extension of the residence time of the dosage form at the site of administration. Gelatin was chosen as another ingredient for the formulation. It has good gelling properties and is widely used in the food industry [40,41,42]. Gellan gum was used due to its swelling ability and anionic properties, allowing for the interaction with ions present in saliva and the cationic active pharmaceutical ingredient (API) [43,44]. GG with a high acylation degree was chosen due to the possibility of obtaining flexible and elastic films [45], while according to the literature, low-acyl gellan is more often used to obtain polymer films [36,46] due to its ability to interact more strongly with the ions present in the environment and the ability to independently create more durable gels and films. In this study, HPMC was the main ingredient in the mixtures, and the properties of the films were modified by the addition of a second polymer: gelatin (matrix A) or gellan gum (matrix B).

The amounts of the polymers and plasticizer used in the formulations were selected based on the visual and mechanical properties of the films. The parameters of the process were optimized to obtain transparent, smooth films that could be easily separated from the mold and further processed. In order to determine the best quantitative composition for matrix A, four polymer films with different compositions were prepared (F1–F4, Table 1). The polymer film containing only HPMC (Table 1, F1) showed high hardness and brittleness, which made it impossible to completely separate it from the glass plate. In the film containing the addition of gelatin at a concentration of 1.5% (F4), turbidity was visible in relation to the mixture before drying. HPMC films with the addition of 0.5% (F2) and 1% gelatin (F3) were flexible and transparent and could be successfully separated from the glass plate. The formulation with the addition of 1% gelatin was selected for further tests due to its greater hardness and elasticity, and it was called matrix A for further investigation.

Next, 10.0 g, 15.0 g and 20.0 g of F3 were poured onto glass plates in order to determine the optimal thickness of the film. The films formed after drying a 10.0 g amount of the solution were thin, delicate and easily torn when separated from the glass plate. The films obtained from 15 g of the solution showed greater strength than the previous ones, while the samples obtained from 20 g of the solution had the greatest thickness, the best strength and elasticity, and the least susceptibility to tearing when separated from the plates. A similar assessment was performed for the polymer films with the addition of GG instead of gelatin. Four mixtures were prepared with the same content of HPMC and a variable concentration of GG (Table 1, F5–F8). Solutions containing 1.0%, 0.5% and 0.1% of gellan were too viscous, which made them difficult to mix, and prevented the solution from being evenly poured into the mold. A mixture containing 0.05% of gellan gum was selected for further testing (F8, Table 1), and was called matrix B. Moreover, the matrices containing GG had the tendency to form bubbles; thus, using a mechanical stirrer was eliminated. Immediately after obtaining a homogeneous solution, the sample was transferred to an ultrasonic bath. Due to the faster gelation of the polymer solution compared to the mixture containing HPMC and gelatin, the sample residence time in the ultrasonic bath was shortened to 11 min. Moreover, the bath was filled with water at a temperature of 90 °C to delay gel solidification. It was also found that the 24 h drying time used in the case of matrix A was insufficient to form the films, so it was extended to 48 h while maintaining the same temperature. The polymer films obtained based on the developed composition and optimized conditions were transparent, flexible and easily separated from the mold. They did not show significant differences in visual assessment. The active substance was successfully incorporated into both matrices, A(CHX) and B(CHX), and its presence did not affect the appearance of the films. The compositions of the prepared films with active substances are presented in Table 2.

### 2.2. Water Loss after Drying

The water loss analysis showed that matrix B lost slightly more water than matrix A (Table 3). This may be due to the longer drying time used in the case of matrix B. The modification in drying time was necessary because, in the case of matrix B, drying for 24 h did not allow us to obtain a homogeneous film with good mechanical strength that was easily separated from the mold.

It should be emphasized that the observed differences in the water content were statistically insignificant, and the products obtained after two different drying times did not differ visually.

### 2.3. Swelling Analysis

**Figure 1:**
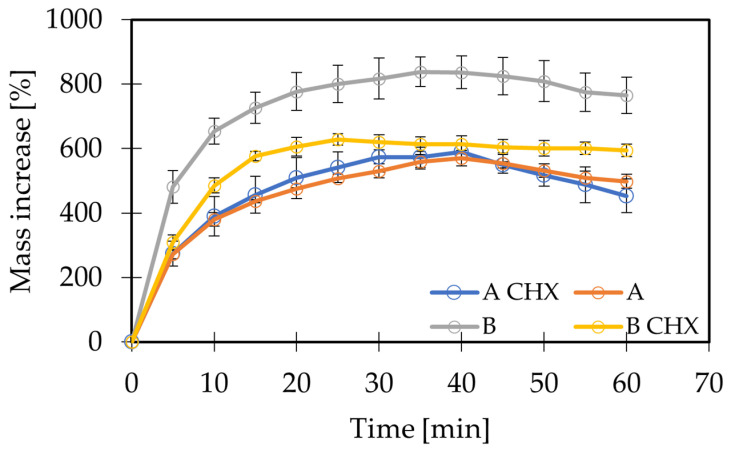
Swelling test for matrices A and B in water.

**Figure 2:**
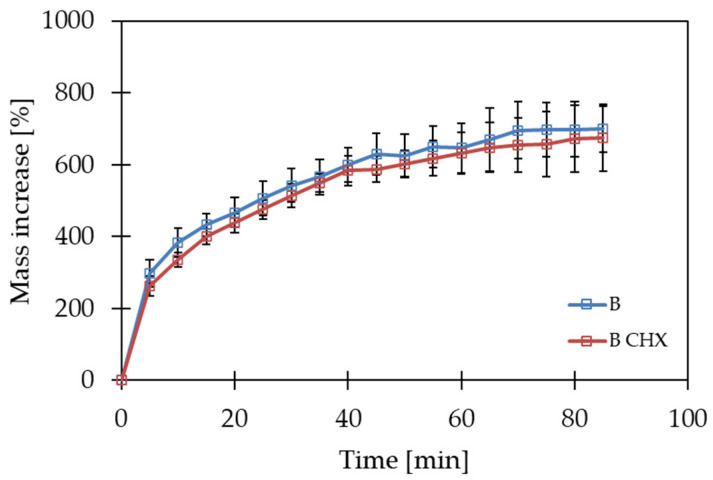
Swelling test of matrix B in artificial saliva.

The aim of this study was to determine the water absorption capacity of polymer films in water or artificial saliva. Due to the type of components forming polymer matrices, it can be assumed that the swelling phase associated with the diffusion of water into the matrix and increasing its volume will only constitute the initial stage of the processes observed after placing the films in an aqueous environment. Due to the good solubility of all the ingredients used in water, dissolution or erosion of the matrix can be expected in the final phase, which will result in a loss of film mass. In the first step, the test was carried out using deionized water. The behavior of matrices A and B was analyzed, both with and without the active substance incorporated. In the next stage, a swelling test in artificial saliva was also performed for polymer films containing GG, due to the anionic nature of the polymer and the possibility of interaction with ions present in the environment of artificial saliva [47]. The obtained results of the swelling tests for matrices A and B in an aqueous environment are presented in Figure 1. The test results for matrix B in artificial saliva are presented in Figure 2.

In water, matrices A and B increased their mass with higher values of percentage mass increase achieved by matrix B. According to the hypothesis presented above, the characteristic profile of the obtained curves indicates a two-stage course of the observed process. In both cases, the mass increase associated with matrix swelling was observed first. The largest mass increase for both matrices occurred during the first 5 min of the test. In the following minutes, the value increased, reaching a peak value, and then decreased, which was probably related to film dissolution. The maximum value of weight gain expressed as a percentage was 837.7% for matrix B and 570.2% for matrix A. Also, at 60 min, the percentage of weight gain for matrix B was greater than that for matrix A, at 764.9% and 498.2%, respectively. In addition, the time to achieve the maximum value was less for matrix B (35 min), while for matrix A it was 40 min. The results showed that the combination of gellan gum and HPMC forms films with higher swelling capacity compared to the mixture of HPMC and gelatin. Matrix B was capable of binding more water than matrix A in a similar time.

A study by Tedesco et al. [48] suggests a lack of interaction between gelatin and hypromellose chains. The opposite phenomenon occurs with GG. GG is a polymer that, due to its anionic nature, can interact with ions present in the environment [43,44]. Thus, an additional swelling test was performed for matrix B in artificial saliva (AS) medium, in which monovalent ions were present (Figure 2). The maximum value of weight gain for matrix B in artificial saliva was 698.7% and was reached after 85 min of the test. The greatest weight gain was observed, as in the case of swelling in an aqueous environment, during the first five minutes. In the artificial saliva environment, however, this increment for matrix B was smaller, as was the maximum value of the weight gain. In addition, the time required to reach the maximum value was much longer. This phenomenon can be explained with the gelation process of GG. When the polymer is heated, there is a change in its structure and the formation of double helixes. These helices can bond together through divalent cations to form more complex structures, while monovalent cations enhance gelation through electrostatic interactions [45]. Monovalent cations are present in artificial saliva. It can be assumed that a tighter gel structure will absorb less water and, consequently, swell more slowly, since a gel layer with higher viscosity is formed at the interface after the polymer comes into contact with the solvent. It forms a barrier with the thickness and viscosity affecting the rate of water penetration inside the matrix. It is also related to the stability of the matrix, since the slower the water penetrates inside the matrix and the chains hydrate, the later the matrix will disintegrate [49]. On the other hand, according to the literature, the high-acyl gellan used for matrix B tends to form soft, weak gels. Moreover, in the presence of salts such as sodium chloride and mucin, which are components of artificial saliva, a decrease in the viscosity of gels whose only component is GG with a high degree of acylation is observed [50]. However, it should be noted that in this study a mixture of two polymers was used, which may affect the nature of the interaction with the components of artificial saliva. Moreover, the aforementioned literature studies used gels obtained by ionotropic gelation with calcium ions, which play an important role in the crosslinking of GG [43,47]. In this study, complex systems obtained without the participation of divalent cations were used, so it can be assumed that their interaction with the components of artificial saliva will be different. The swelling test was also carried out for matrices with incorporated active substances. Analogously, the swelling test was carried out for matrix A in water and for matrix B in water and artificial saliva. For the A(CHX) matrix, the swelling process in water was similar to that for the matrix without the active ingredient (Figure 1, blue line). For both systems, similar values of percentage weight gain were observed at the end of the test. On the contrary, in the case of matrix B(CHX), this study showed lower swelling values and a flatter curve compared to matrix B. In contrast, the B(CHX) matrix tested in artificial saliva behaved similarly to the B matrix in this environment. The values of percentage weight gain at the end of the study (85 min) showed no significant difference. It can be speculated that the cationic chlorhexidine contained in the B(CHX) matrix affected the weight gain by interacting with GG. On the other hand, in the artificial saliva environment, in the presence of monovalent ions, both matrices behaved similarly, which may indicate a stronger interaction occurring between gellan gum and ions in solution than with chlorhexidine. The swelling tests of matrices in water allowed us to conclude that all the tested films in contact with the aqueous environment swell in the first stage, which is manifested by an increase in the weight of the samples, while in the next stage the polymer matrix is dissolved or eroded. For drug-free matrices tested in water, the swelling was stronger in the case of films containing gellan gum when compared to those with the addition of gelatin. These differences were also observed for the chlorhexidine-containing systems. In matrix A, the addition of CHX did not affect the swelling test results significantly; however, the difference was visible in the case of matrix B. It is worth noting that in a study conducted in an artificial saliva environment, no differences were found in the behavior of the B matrices with and without the drug (Figure 2). This may mean that the presence of monovalent ions in the environment was the decisive factor affecting the swelling process of the films. The results suggest that the use of GG as a component of the polymer matrix in oral films may contribute to prolonging the film disintegration process, which may simultaneously affect the release of the active substance.

### 2.4. Disintegration Time

Disintegration/erosion time tests were performed for all matrices in water and additionally for matrix B in artificial saliva (AS). The results are presented in Table 4.

The matrices containing a mixture of HPMC and gelatin showed lower disintegration times than the matrices with HPMC and gellan gum, which can be correlated with stronger swelling by the formulations with GG. Trastullo et al. [51] conducted a study using matrices obtained by casting from HPMC and gelatin mixtures using similar amounts of polymers and obtained films that disintegrated in several dozen minutes. Tedesco et al. [48] conducted similar research, but they used type-E hypromellose to produce matrices and obtained films that disintegrated in less than a minute. This suggests a significant influence of the type of HPMC on the durability of the matrix and, consequently, the possibility of influencing the effect of prolonged drug release by the appropriate selection of the type of polymer. According to available literature data, K-type hypromellose is a better choice for the production of matrices intended for prolonged drug release [36,52]. The longest disintegration/erosion time was observed for the B(CHX) matrix (Table 4). No disintegration was observed for B(CHX) in both the aqueous environment and artificial saliva within 180 min of testing. Studies in the literature describing systems containing GG and chlorhexidine mostly refer to low-acetylated gellan gum and a different type of HPMC than the one used in this study. Gandhi et al. [53] developed gellan-based matrices with a low degree of acylation containing CHX. In their research, they confirmed the lack of interaction and compatibility of both ingredients, but a comparison of the disintegration time of matrices with and without the incorporated drug was not carried out. Matrices obtained using GG with a low degree of acylation in water or artificial saliva did not disintegrate within a few to several hours, depending on the polymer concentration [53,54]. It should be emphasized that the polymer concentrations used in the studies described above were several times higher than the concentration used in this study. Matrix B without the incorporated drug in both environments showed lower durability compared to matrix B(CHX), while in water it turned out to be more durable than in artificial saliva. Observations can be related to these from swelling studies. Matrix B in water had greater swelling capacity and reached a greater mass in a shorter time than in artificial saliva. Assuming that the dissolution rate of both matrices was similar, it can be concluded that erosion took longer due to the greater mass of the sample after swelling. All matrices remained in place until they were completely eroded. It should be emphasized, however, that the in vitro test performed does not fully reflect the conditions in the oral cavity. Additional factors, such as mechanical abrasion associated with eating and speaking, may reduce the residence time of the film at the application site.

### 2.5. Hygroscopicity Study

**Figure 3:**
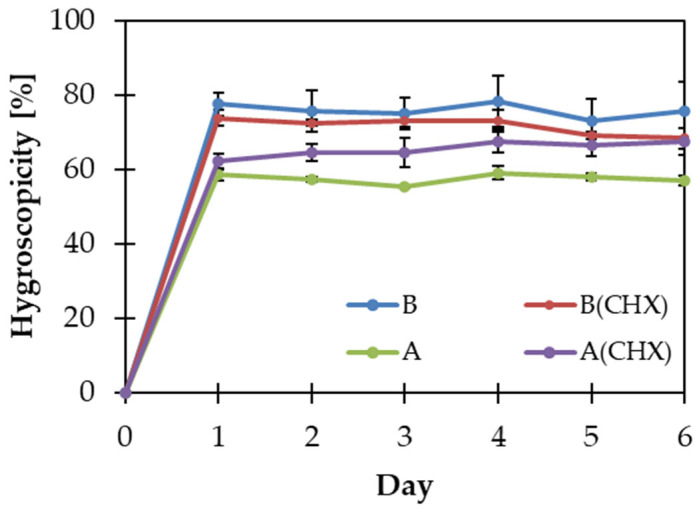
Change in matrix hygroscopicity over 6 days of the study.

The results of the hygroscopicity test presented as a percentage increase in the sample mass with time are shown in Figure 3.

The weight of all matrices increased on the first day of the study. A mass increase of more than 15% in 24 h indicates high hygroscopicity [55], and all matrices met this criterion. Matrix B showed significantly higher hygroscopicity than matrix A at the end of the study, which may confirm the influence of gellan on increasing the water-binding capacity of the matrix. However, there was no significant difference between matrices with and without the addition of API. The study was conducted for 6 consecutive days to observe changes in the weight of the films. In all cases, the polymer films increased in mass during the first 24 h. After that time, their masses changed slightly. This means that within 24 h, an amount of water was absorbed sufficient to saturate the matrices.

The results obtained provide information that may be useful when selecting packaging and storing the obtained films. The high ability to absorb water from the atmosphere indicates the need to store the matrices in tightly closed individual packages or to use a desiccant in collective packages.

### 2.6. Tensile Strength

The force needed to break the polymer film and the film elongation at the moment of breaking (defined as the value of the displacement of the upper part of the texturometer attachment in relation to the initial position) for all tested samples are presented in Table 5.

Matrix A showed the highest mechanical strength. According to studies available in the literature [48,56], HPMC itself forms flexible polymer films, but the presence of gelatin affects the mechanical properties of the film, making it more hard and durable. However, the lower mechanical strength of matrix B may be due to the fact that its composition includes K-type HPMC and GG with high acylation, i.e., two polymers with the ability to create delicate gels and films [36,45]. Matrices containing the active substance turned out to be less durable than their counterparts without the drug, which may indicate an interaction of the active substance with the matrix components. Matrix A, despite its greater hardness, presented similar displacement values to matrix B. Similar conclusions can be drawn in the case of matrices with API.

It is important to notice that the therapeutic approach involving both drug delivery and wound healing actions is relatively new and the studies describing these formulations are rare, while most of the literature reports focus on fast dissolving oral films considered as an alternative to conventional oral tablets or capsules. In these formulations, usually different types of HPMC, different polymer content and also the addition of other excipients, like for examples disintegrants, are applied in order to increase the disintegration rate. In order to obtain polymer films revealing prolonged residence time at the affected site in oral cavity with prolonged drug delivery, the selected matrix-forming polymer must display appropriate swelling ability. The systems with the mentioned characteristics were obtained and described by Campos et al. [28], however, the matrices investigated in the study contained the addition of polyacrylic acid, sodium hyaluronate and nanoparticles. The comparison between the literature reports [28,48,57] and the findings of this study indicate that the viscosity of the applied HPMC is crucial for the type and the properties of the obtained dosage forms. In the case of the formulations disintegrating quickly upon the contact with saliva, low viscosity grade HPMC, sometimes with additional disintegration agents, is required. In formulations designed for an extended stay in the oral cavity, higher polymer viscosity is necessary. 

## 3. Conclusions

Analyzing the results obtained during this study, it can be concluded that the mixture of HPMC and gellan gum or gelatin could be used to prepare transparent, flexible polymer films. The active substance was successfully incorporated into both types of matrices and did not change the appearance of the films. Based on swelling tests, a two-stage course of this process was found. In the first phase, the matrix swelled as a result of water diffusion into its interior, while in the second phase, its dissolution/erosion took place. Also, polymer films containing a mixture of HPMC and gellan gum (matrix B) showed greater swelling capacity than films containing HPMC and gelatin (matrix A). Both types of matrices disintegrated at a similar rate; however, due to its greater swelling capacity, matrix B had a longer disintegration time in an aqueous environment. Also, matrix B containing gellan gum had a lower swelling capacity in artificial saliva than in water. This indicates a possible interaction with the components of artificial saliva, resulting in the formation of a layer inhibiting further penetration of water into the matrix. The presence of chlorhexidine in matrix B also reduced its swelling ability in water, which was not observed in the artificial saliva environment. On the basis of the disintegration/erosion tests, it was found that this process is slower in the case of matrix B, which may be related to the greater weight gain during swelling. Also, this study showed that in water and artificial saliva, the B matrices with the incorporated drug were stable for more than 3 h, unlike the analogous A matrices. This allows us to assume that the composition of the B matrices is more suitable for obtaining the preparation with prolonged stay at the site of administration. All tested films showed high hygroscopicity, absorbing enough water from the air to saturate the matrix within 24 h. At the end, a relationship was found between the composition of polymer films and their mechanical strength. Matrix A containing the addition of gelatin showed greater strength compared to matrix B. Also, the presence of chlorhexidine influenced the mechanical strength of the matrices. The value of the force necessary to tear the film and the tensile strength decreased under the influence of the drug.

Performed studies allowed for the development and optimization of the formulation and manufacturing process of polymer films. We also conducted preliminary studies to investigate parameters such as water loss, swelling ability, disintegration time, and hygroscopicity of the samples. Moreover, a basic mechanical analysis was performed. These studies help predict prepared polymeric films’ performance during application and storage conditions. It should be emphasized that it is challenging to fully mimic the conditions in the oral cavity. However, the processes such as swelling and disintegration naturally occur at the application site. Also, the hygroscopicity study helps choose the suitable packaging for the product. Mechanical studies could be used to assess both the packaging conditions and resistance to damage during application. 

In follow-up steps, the safety and efficiency of the films should be examined. Further studies should involve the in vitro release tests to assess the drug release profile. Next, in vivo studies should be performed. The test of film residence time on buccal mucosa could be conducted on human volunteers to obtain data on bioadhesive properties and residence time of the film after application in a destined environment. Such a test was described by Peh and Wong [58]. However, in this study, the volunteers were restricted from eating, drinking, and talking during the study. This approach could be modified to better mimic the actual behavior of the films, e.g., subjects could be asked to drink a certain amount of water or allowed to talk freely over a specific time. Another interesting in vivo test was conducted by Juliano et al. [59] on three healthy human volunteers. Oral films loaded with chlorhexidine were placed on the buccal mucosa, and at specific time points, the saliva samples were taken, and the drug concentration was measured. This study could also bring information on any eventual irritation or inconvenience. However, it could be beneficial to increase the number of tested subjects.

Further studies on our formulations are necessary and certainly will lead to obtaining more data to develop safe and effective products. 

## 4. Materials and Methods

Gellan gum CG-HA and hypromellose (HPMC) Metolose® 90SH-4000 were kindly donated free of charge by CP Kelco (Atlanta, GA, USA) and Shin-Etsu (Tokyo, Japan), respectively. Food-grade gelatin was purchased from a local store. Glycerol 85% and chlorhexidine digluconate (CHX) were purchased from Fagron (Rotterdam, Netherlands). The artificial saliva was prepared using the following reagents: sodium chloride (POCH, Gliwice, Poland), potassium chloride (POCH, Gliwice, Poland), potassium dihydrogen phosphate (Chempur, Piekary Śląskie, Poland), urea (PPH Galfarm, Kraków, Poland), sodium bicarbonate (PoCH, Gliwice, Poland), and mucin (Sigma-Aldrich, St Louis, MO, USA). All reagents except for the commercially available gelatin were of analytical grade and were used without further purification.

### 4.1. Polymer Film Preparation

To select the appropriate quantitative composition, four types of polymer products were prepared and visually assessed (F1–F4, Table 1). The composition of each formulation is presented in Table 1. Accurate amounts of glycerol and water followed by gelatin and HPMC were added into a beaker with a magnetic stirrer. The mixture was heated under a cover to a temperature of 80 ± 5 °C and mixed simultaneously with a magnetic stirrer to obtain a homogeneous solution. Next, the solution was mixed using a mechanical stirrer for 30 min at a speed of 500 rpm, and then for another 30 min at a speed of 300 rpm. The obtained solution was subjected to ultrasounds through two cycles of 8 min at room temperature. The foam eventually formed on the surface was removed. Subsequently, the accurate amount of the solution was cast into the 161.12 cm^2^ rectangular silicone mold and dried for 24 h at 40 ± 2 °C. The dried films underwent the visual assessment. The formulation containing 1% of gelatin (matrix A) was chosen for further studies due to it having the most proper visual features and elasticity. The next step was the preparation of HPMC films with the addition of gellan gum instead of gelatin. The solutions containing the same amount of HPMC and various concentrations of the GG (Table 1) were prepared. Accurate amounts of water and glycerol were weighted into a baker. The solution was stirred using a magnetic stirrer and heated to 80 ± 5 °C. Subsequently, the GG and HPMC were added to the heated solution. Clear solution underwent ultrasounds for 11 min at a water temperature of 90 °C. The obtained mixture was poured into a mold with an area of 161.12 cm^2^, previously stored at a temperature of 40 ± 2 °C, and dried for 48 h while maintaining the same temperature. Due to the difficulties in obtaining films containing gellan gum at a concentration of 0.1% and higher, a polymer film containing 0.05% of the GG (matrix B) was selected for further research.

### 4.2. Water Loss after Drying

20 g of HPMC and gelatin solution (solution A) and 23.3 g of HPMC and gellan solution (solution B) were poured onto glass plates of known mass, with three plates per solution. The amount of solution B was selected in such a way as to obtain the same amount of solid ingredients after drying as in the case of solution A and to obtain a similar film thickness. The plates with solution A were placed at a temperature of 40 ± 2 °C for 24 h, and the plates with solution B for 48 h. After drying, the plates with films were weighed and the water loss after drying (Equation (1)) was calculated. The results were assessed with U Mann–Whitney test using Statistica software ver. 13 (TIBCO Software Inc. Palo Alto, CA, USA). The level of significance in the test was set at 0.05.

(1) $$water\;loss=\frac{n_{1}-n_{2}}{n_{1}-n_{3}}\cdot 100\%$$

where n_1_ is the mass of a glass plate with the solution; n_2_ is the mass of a glass plate with a dry film; n_3_ is the mass of an empty glass plate.

### 4.3. Obtaining Film with Chlorhexidine

The amount of added chlorhexidine (CHX) was calculated so that its content in the dry film was 0.1%. Films with the active substance, matrix A(CHX) and matrix B(CHX), were prepared according to the procedure described in Section 4.1, while the previously prepared 1% CHX solution was added to water and glycerol. The compositions of the mixtures prepared to obtain films with the active substance are presented in Table 2.

### 4.4. Characterization of Polymer Films

#### 4.4.1. Swelling Study

Three 25 mL beakers of known mass were prepared. In each beaker, a previously weighed disc of polymer film with a diameter of 19 mm and a thickness of 0.1 mm was placed at the bottom. A total of 10 mL of deionized water or artificial saliva solution (SS) was added to the beakers. The temperature of the solutions was 37.0 ± 0.5 °C, and the composition of SS is given in Table 6 [50].

The beakers were placed in a 37.0 ± 0.5 °C water bath with water for 5 min. After this time, the beakers were removed, the liquid was poured out and the walls of the bakers were thoroughly dried with a paper towel. Each beaker was weighed, a fresh 10 mL of liquid was added to the beakers and they were incubated for another 5 min, measuring the time from the moment the beaker was placed in the water bath. The liquid for replacement was kept at a temperature of 37.0 ± 0.5 °C. The test was carried out for 60 min, as the total residence time of the samples in the solution, or longer, until a constant mass was obtained. The results were assessed with a T test and one-way analysis of variance (ANOVA) for artificial saliva and water, respectively. The calculations were performed with Statistica software ver. 13 (TIBCO Software Inc., Palo Alto, CA, USA). The level of significance in the tests was set at 0.05.

#### 4.4.2. Disintegration Time

The study was performed using a pharmacopoeial apparatus for tablet disintegration time studies PTZ-1E (Pharma Test, Hainburg, Germany). Polymer film discs with a diameter of 19 mm and a thickness of 0.1 mm were placed individually on the walls of glass tubes halfway along their length. Due to their adhesive properties, the samples remained in a specific place and did not require additional fixing. The test was performed in 800 mL of water or artificial saliva at a temperature of 37.0 ± 0.5 °C and a frequency of 30 ± 2 cycles per minute. The time required for all six samples to completely disintegrate or erode was measured. The study was conducted for 3 h.

#### 4.4.3. Hygroscopicity of Films

Polymer film discs with a diameter of 19 mm and a thickness of 0.1 mm were placed individually in screw-on 15 mL Falcon tubes of known mass (*m*_1_). The test tubes with contents (*m*_2_) were weighed and placed without the cap in a temperature and humidity chamber at a temperature of 25 ± 1 °C and a relative humidity of 80 ± 2%. The weight of the samples was measured at 24 h intervals (*m*_3_) for 6 consecutive days. Based on the results obtained, the percentage weight increase in the samples was calculated according to the formula below (Equation (2)) and the level of hygroscopicity was determined [60] according to the classification shown in Table 7.

(2) $$mass\;increase=\frac{m_{3}-m_{2}}{m_{2}-m_{1}}\times 100\%$$

where *m*_1_ is the mass of the empty tube; *m*_2_ is the mass of the tube and sample before study; *m*_3_ is the mass of the tube and sample at subsequent time points.

The results were assessed with an ANOVA test (α = 0.05) performed with Statistica software ver. 13 (TIBCO Software Inc., Palo Alto, CA, USA).

#### 4.4.4. Mechanical Strength

**Figure 4:**
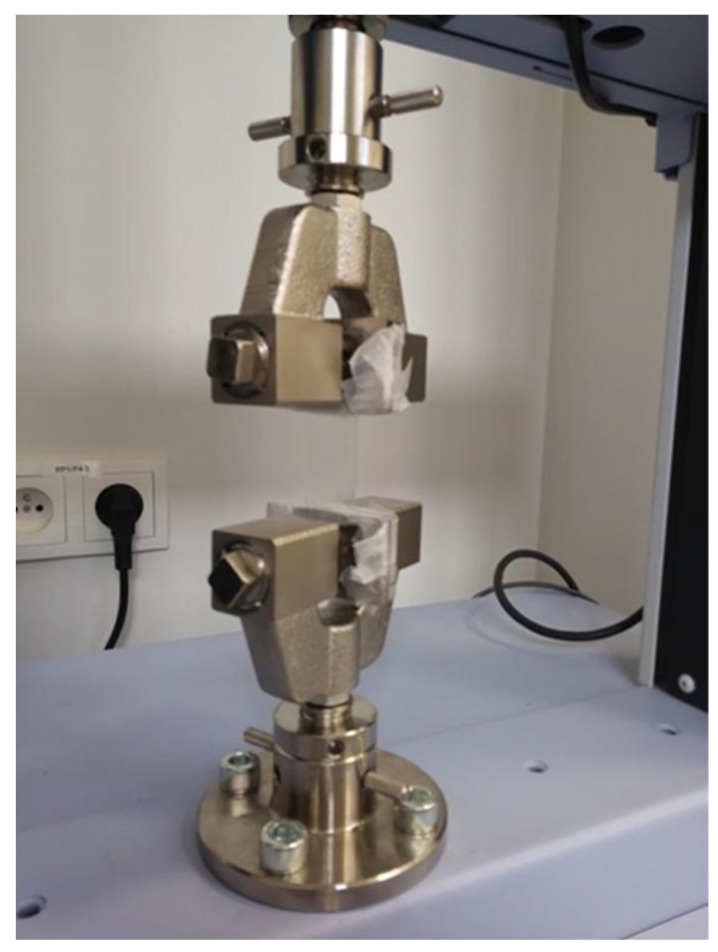
Attachments for the tensile strength study with the film mounted between the clamps.

The test was performed using a Shimadzu AGS-X texture analyzer. Shimadzu tensile test grips were used as attachments (Figure 4). The aim of this study was to determine the tensile strength of polymer films and to assess their elastic properties. A rectangle 50 mm long and 25 mm wide was cut out of a 0.1 mm thick polymer film. The sample was placed between the apparatus attachments and its shorter edges were inserted 10 mm into each grip. The elements of the grips remaining in contact with the film were wrapped with a layer of parafilm to protect the sample from being cut by a sharp edge. Due to the small thickness and smooth surface of the sample, the presence of parafilm also contributed to its better fixation, preventing the sample from sliding out of the holder during the test. The dimensions of the part of the patch tested were a 30 mm length and 25 mm width. The upper attachment was moved vertically upwards at a speed of 10 mm/min. The test was carried out until the sample cracked. The value of the force acting on the film at the moment of breakage and the corresponding displacement of the upper movable part of the attachment relative to the lower one were recorded. The results were analyzed with ANOVA tests (α = 0.05) performed with Statistica software ver. 13 (TIBCO Software Inc., Palo Alto, CA, USA).

## Figures and Tables

**Table 1:** The composition of formulations (solutions) for the preliminary assessment of polymer films with HPMC and gelatin or gellan gum additive. Formulations 3 and 8 were selected for further studies (matrices A and B, respectively).

Formulation	HPMC [%, *w*/*w*]	Gelatin [%, *w*/*w*]	Gellan Gum [%, *w*/*w*]	Glycerol [%, *w*/*w*]	Water [%, *w*/*w*]
F1	1.5	-	-	-	98.5
F2	1.5	0.5	-	5.0	93.0
F3	1.5	1.0	-	5.0	92.5
F4	1.5	1.5	-	5.0	92.0
F5	1.5	-	1	5.0	92.5
F6	1.5	-	0.5	5.0	93.0
F7	1.5	-	0.1	5.0	93.4
F8	1.5	-	0.05	5.0	93.45

**Table 2:** The composition of solutions containing the active substance for the film preparation.

Formulation	HPMC [%, *w*/*w*]	Gelatin [%, *w*/*w*]	Gellan Gum [%, *w*/*w*]	Glycerol [%, *w*/*w*]	1% CHX Solution [%, *w*/*w*]	Water [%, *w*/*w*]
A(CHX)	1.5	1.0	-	5.0	0.7	92.50
B(CHX)	1.5	-	0.05	5.0	0.63	92.82

**Table 3:** Water loss and the amount of water in dried polymeric films.

Formulation	Water Loss after Drying [%] *
A	92.94 ± 0.04
B	93.71 ± 0.50

*: in relation to the initial water content in the solution.

**Table 4:** Disintegration time of the matrices in water and artificial saliva (AS).

Sample	Disintegration Time [min]
A	106 ± 9
A(CHX)	77 ± 5
B	170 ± 17
B(CHX)	>180
B (AS)	152 ± 16
B(CHX) (AS)	>180

**Table 5:** The values of force and displacement at the moment of film breaking.

Type of Film	Force [N]	Displacement [mm]	Elongation at Break [%]
A	33.2 ± 2.4	24.0 ± 2.3	180.0 ± 7.7
A(CHX)	15.5 ± 1.9	14.8 ± 1.5	149.3 ± 5.0
B	22.9 ± 0.7	20.0± 2.4	166.7 ± 8.0
B(CHX)	8.7 ± 2.0	15.8 ± 2.8	152.7 ± 9.3

**Table 6:** The composition of the artificial saliva solution [50].

Ingredient	Amount [g]
NaCl	0.125
KCl	0.964
KH_2_PO_4_	0.654
Urea	0.2
NaHCO_3_	0.631
Mucin	10.0
Water	ad 1000.0

**Table 7:** Classification of materials in terms of the degree of hygroscopicity.

Hygroscopicity	Mass Increase
Soluble under the influence of air humidity	The sample absorbs moisture until it dissolves in the absorbed water
Large	≥15%
Medium	≥2% and <15%
Small	≥0.2% and <2%

## Data Availability

The data supporting the reported results can be obtained from the correspondence author.

## References

[1] Shetty S.S., Maruthi M., Dhara V., de Arruda J.A.A., Abreu L.G., Mesquita R.A., Teixeira A.L., Silva T.A., Merchant Y. (2022). Oral Mucositis: Current Knowledge and Future Directions. Dis. -A-Mon..

[2] Sedghi L., DiMassa V., Harrington A., Lynch S.V., Kapila Y.L. (2021). The Oral Microbiome: Role of Key Organisms and Complex Networks in Oral Health and Disease. Periodontology 2000.

[3] Watanabe Y., Okada K., Kondo M., Matsushita T., Nakazawa S., Yamazaki Y. (2020). Oral Health for Achieving Longevity. Geriatr. Gerontol. Int..

[4] Tiensripojamarn N., Lertpimonchai A., Tavedhikul K., Udomsak A., Vathesatogkit P., Sritara P., Charatkulangkun O. (2021). Periodontitis Is Associated with Cardiovascular Diseases: A 13-Year Study. J. Clin. Periodontol..

[5] Bengtsson V.W., Persson G.R., Berglund J.S., Renvert S. (2021). Periodontitis Related to Cardiovascular Events and Mortality: A Long-Time Longitudinal Study. Clin. Oral Investig..

[6] Kurtzman G.M., Horowitz R.A., Johnson R., Prestiano R.A., Klein B.I. (2022). The Systemic Oral Health Connection: Biofilms. Medicine.

[7] Sanz M., Marco del Castillo A., Jepsen S., Gonzalez-Juanatey J.R., D’Aiuto F., Bouchard P., Chapple I., Dietrich T., Gotsman I., Graziani F. (2020). Periodontitis and Cardiovascular Diseases: Consensus Report. J. Clin. Periodontol..

[8] Bélanger M., Kozarov E., Song H., Whitlock J., Progulske-Fox A. (2012). Both the Unique and Repeat Regions of the *Porphyromonas Gingivalis* Hemagglutin A Are Involved in Adhesion and Invasion of Host Cells. Anaerobe.

[9] Naderi S., Merchant A.T. (2020). The Association Between Periodontitis and Cardiovascular Disease: An Update. Curr. Atheroscler. Rep..

[10] Martínez-García M., Hernández-Lemus E. (2021). Periodontal Inflammation and Systemic Diseases: An Overview. Front. Physiol..

[11] Kikutani T., Tamura F., Tashiro H., Yoshida M., Konishi K., Hamada R. (2015). Relationship between Oral Bacteria Count and Pneumonia Onset in Elderly Nursing Home Residents. Geriatr. Gerontol. Int..

[12] Shay K. (2002). Infectious Complications of Dental and Periodontal Diseases in the Elderly Population. Clin. Infect. Dis..

[13] Pulito C., Cristaudo A., Porta C.L., Zapperi S., Blandino G., Morrone A., Strano S. (2020). Oral Mucositis: The Hidden Side of Cancer Therapy. J. Exp. Clin. Cancer Res..

[14] Trotti A., Bellm L.A., Epstein J.B., Frame D., Fuchs H.J., Gwede C.K., Komaroff E., Nalysnyk L., Zilberberg M.D. (2003). Mucositis Incidence, Severity and Associated Outcomes in Patients with Head and Neck Cancer Receiving Radiotherapy with or without Chemotherapy: A Systematic Literature Review. Radiother. Oncol..

[15] Kusiak A., Jereczek-Fossa B.A., Cichońska D., Alterio D. (2020). Oncological-Therapy Related Oral Mucositis as an Interdisciplinary Problem—Literature Review. Int. J. Environ. Res. Public Health.

[16] Elad S., Cheng K.K.F., Lalla R.V., Yarom N., Hong C., Logan R.M., Bowen J., Gibson R., Saunders D.P., Zadik Y. (2020). MASCC/ISOO Clinical Practice Guidelines for the Management of Mucositis Secondary to Cancer Therapy. Cancer.

[17] González-Moles M.Á., Warnakulasuriya S., González-Ruiz I., González-Ruiz L., Ayén Á., Lenouvel D., Ruiz-Ávila I., Ramos-García P. (2021). Worldwide Prevalence of Oral Lichen Planus: A Systematic Review and Meta-Analysis. Oral Dis..

[18] Cui R.Z., Bruce A.J., Rogers R.S. (2016). Recurrent Aphthous Stomatitis. Clin. Dermatol..

[19] Yogarajah S., Setterfield J. (2021). Mouth Ulcers and Diseases of the Oral Cavity. Medicine.

[20] Jain N., Dutt U., Radenkov I., Jain S. (2024). WHO’s Global Oral Health Status Report 2022: Actions, Discussion and Implementation. Oral Dis..

[21] Poppolo Deus F., Ouanounou A. (2022). Chlorhexidine in Dentistry: Pharmacology, Uses, and Adverse Effects. Int. Dent. J..

[22] Bescos R., Ashworth A., Cutler C., Brookes Z.L., Belfield L., Rodiles A., Casas-Agustench P., Farnham G., Liddle L., Burleigh M. (2020). Effects of Chlorhexidine Mouthwash on the Oral Microbiome. Sci. Rep..

[23] Puig-Asensio M., Marra A.R., Childs C.A., Perencevich E.N., Schweizer M.L. (2020). Chlorhexidine Dressings to Prevent Catheter-Related Bloodstream Infections: A Systematic Literature Review and Meta-Analysis. Infect. Control Hosp. Epidemiol..

[24] Azzopardi A., Trapani J. (2024). Chlorhexidine-Based versus Non-Chlorhexidine Dressings to Prevent Catheter-Related Bloodstream Infections: An Evidence-Based Review. Nurs. Crit. Care.

[25] Cheong J.Z.A., Liu A., Rust C.J., Tran C.L., Hassan S.E., Kalan L.R., Gibson A.L.F. (2022). Robbing Peter to Pay Paul: Chlorhexidine Gluconate Demonstrates Short-Term Efficacy and Long-Term Cytotoxicity. Wound Repair Regen..

[26] Van den Poel B., Saegeman V., Schuermans A. (2022). Increasing Usage of Chlorhexidine in Health Care Settings: Blessing or Curse? A Narrative Review of the Risk of Chlorhexidine Resistance and the Implications for Infection Prevention and Control. Eur. J. Clin. Microbiol. Infect. Dis..

[27] Steinberg D., Friedman M. (2020). Sustained-Release Delivery of Antimicrobial Drugs for the Treatment of Periodontal Diseases: Fantasy or Already Reality?. Periodontology 2000.

[28] Campos J.C., Cunha D., Ferreira D.C., Reis S., Costa P.J. (2021). Oromucosal Precursors of *in Loco* Hydrogels for Wound-Dressing and Drug Delivery in Oral Mucositis: Retain, Resist, and Release. Mater. Sci. Eng. C.

[29] Nafee N.A., Ismail F.A., Boraie N.A., Mortada L.M. (2004). Mucoadhesive Delivery Systems. I. Evaluation of Mucoadhesive Polymers for Buccal Tablet Formulation. Drug Dev. Ind. Pharm..

[30] Ahmady A., Abu Samah N.H. (2021). A Review: Gelatine as a Bioadhesive Material for Medical and Pharmaceutical Applications. Int. J. Pharm..

[31] Szekalska M., Citkowska A., Wróblewska M., Winnicka K. (2021). The Impact of Gelatin on the Pharmaceutical Characteristics of Fucoidan Microspheres with Posaconazole. Materials.

[32] Bonferoni M.C., Chetoni P., Giunchedi P., Rossi S., Ferrari F., Burgalassi S., Caramella C. (2004). Carrageenan–Gelatin Mucoadhesive Systems for Ion-Exchange Based Ophthalmic Delivery: In Vitro and Preliminary In Vivo Studies. Eur. J. Pharm. Biopharm..

[33] Li A., Khan I.N., Khan I.U., Yousaf A.M., Shahzad Y. (2021). Gellan Gum-Based Bilayer Mucoadhesive Films Loaded with Moxifloxacin Hydrochloride and Clove Oil for Possible Treatment of Periodontitis. Drug Des. Dev. Ther..

[34] de Oliveira Cardoso V.M., de Brito N.A.P., Ferreira N.N., Boni F.I., Ferreira L.M.B., Carvalho S.G., Gremião M.P.D. (2021). Design of Mucoadhesive Gellan Gum and Chitosan Nanoparticles Intended for Colon-Specific Delivery of Peptide Drugs. Colloids Surf. A Physicochem. Eng. Asp..

[35] Gadziński P., Froelich A., Jadach B., Wojtyłko M., Tatarek A., Białek A., Krysztofiak J., Gackowski M., Otto F., Osmałek T. (2023). Ionotropic Gelation and Chemical Crosslinking as Methods for Fabrication of Modified-Release Gellan Gum-Based Drug Delivery Systems. Pharmaceutics.

[36] Borges A.F., Silva C., Coelho J.F.J., Simões S. (2015). Oral Films: Current Status and Future Perspectives. J. Control. Release.

[37] Dixit R.P., Puthli S.P. (2009). Oral Strip Technology: Overview and Future Potential. J. Control. Release.

[38] Ding C., Zhang M., Li G. (2015). Preparation and Characterization of Collagen/Hydroxypropyl Methylcellulose (HPMC) Blend Film. Carbohydr. Polym..

[39] Ruel-Gariépy E., Leroux J.-C. (2004). In Situ-Forming Hydrogels—Review of Temperature-Sensitive Systems. Eur. J. Pharm. Biopharm..

[40] Kowalski Z., Banach M., Makara A. (2011). Preparation of Low Temperature Strongly Gelling Protein (Gelatine) by Chemical Methods. Chemik.

[41] Foox M., Zilberman M. (2015). Drug Delivery from Gelatin-Based Systems. Expert Opin. Drug Deliv..

[42] Gómez-Guillén M.C., Giménez B., López-Caballero M.E., Montero M.P. (2011). Functional and Bioactive Properties of Collagen and Gelatin from Alternative Sources: A Review. Food Hydrocoll..

[43] Morris E.R., Nishinari K., Rinaudo M. (2012). Gelation of Gellan—A Review. Food Hydrocoll..

[44] Kirchmajer D.M., Steinhoff B., Warren H., Clark R., in het Panhuis M. (2014). Enhanced Gelation Properties of Purified Gellan Gum. Carbohydr. Res..

[45] Zia K.M., Tabasum S., Khan M.F., Akram N., Akhter N., Noreen A., Zuber M. (2018). Recent Trends on Gellan Gum Blends with Natural and Synthetic Polymers: A Review. Int. J. Biol. Macromol..

[46] Ismail N.A., Amin K.A.M., Razali M.H. (2018). Preparation of Gellan Gum (GG) Film: The Effect of GG, Calcium Chloride (CaCl_2_), Glycerol Concentration and Heat Treatment. IOP Conf. Ser. Mater. Sci. Eng..

[47] Swain G.P., Patel S., Gandhi J., Shah P. (2019). Development of Moxifloxacin Hydrochloride Loaded In-Situ Gel for the Treatment of Periodontitis: In-Vitro Drug Release Study and Antibacterial Activity. J. Oral Biol. Craniofac. Res..

[48] Tedesco M.P., Monaco-Lourenço C.A., Carvalho R.A. (2016). Gelatin/Hydroxypropyl Methylcellulose Matrices—Polymer Interactions Approach for Oral Disintegrating Films. Mater. Sci. Eng. C.

[49] Colombo P., Bettini R., Santi P., Peppas N.A. (2000). Swellable Matrices for Controlled Drug Delivery: Gel-Layer Behaviour, Mechanisms and Optimal Performance. Pharm. Sci. Technol. Today.

[50] Osmałek T.Z., Froelich A., Jadach B., Krakowski M. (2018). Rheological Investigation of High-Acyl Gellan Gum Hydrogel and Its Mixtures with Simulated Body Fluids. J. Biomater. Appl..

[51] Trastullo R., Abruzzo A., Saladini B., Gallucci M.C., Cerchiara T., Luppi B., Bigucci F. (2016). Design and Evaluation of Buccal Films as Paediatric Dosage Form for Transmucosal Delivery of Ondansetron. Eur. J. Pharm. Biopharm..

[52] Salunke S.R., Patil S.B. (2016). Ion Activated in Situ Gel of Gellan Gum Containing Salbutamol Sulphate for Nasal Administration. Int. J. Biol. Macromol..

[53] Gandhi K. (2017). Formulation and Evaluation of Sol-Gel Drug Delivery System for Intracanal pH Sensitive Controlled Delivery of Chlorhexidine. JCDR.

[54] Paolicelli P., Petralito S., Varani G., Nardoni M., Pacelli S., Di Muzio L., Tirillò J., Bartuli C., Cesa S., Casadei M.A. (2018). Effect of Glycerol on the Physical and Mechanical Properties of Thin Gellan Gum Films for Oral Drug Delivery. Int. J. Pharm..

[55] Allada R., Maruthapillai A., Palanisamy K., Chappa P. (2016). Hygroscopicity Categorization of Pharmaceutical Solids by Gravimetric Sorption Analysis: A Systematic Approach. Asian J. Pharm..

[56] Liew K.B., Tan Y.T.F., Peh K.-K. (2014). Effect of Polymer, Plasticizer and Filler on Orally Disintegrating Film. Drug Dev. Ind. Pharm..

[57] Adrover A., Varani G., Paolicelli P., Petralito S., Di Muzio L., Casadei M.A., Tho I. (2018). Experimental and Modeling Study of Drug Release from HPMC-Based Erodible Oral Thin Films. Pharmaceutics.

[58] Peh K.K., Wong C.F. (1999). Polymeric Films as Vehicle for Buccal Delivery: Swelling, Mechanical, and Bioadhesive Properties. J. Pharm. Pharm. Sci..

[59] Juliano C., Cossu M., Pigozzi P., Rassu G., Giunchedi P. (2008). Preparation, In Vitro Characterization and Preliminary In Vivo Evaluation of Buccal Polymeric Films Containing Chlorhexidine. AAPS PharmSciTech.

[60] (2005). Characters Section in Monographs. European Pharmacopeia.

